# Influence of Lactic Acid Bacterium Strains on Changes in Quality, Functional Compounds and Volatile Compounds of Mango Juice from Different Cultivars during Fermentation

**DOI:** 10.3390/foods11050682

**Published:** 2022-02-25

**Authors:** Nobahle P. Cele, Stephen A. Akinola, Vimbainashe E. Manhivi, Tinotenda Shoko, Fabienne Remize, Dharini Sivakumar

**Affiliations:** 1 Phytochemical Food Network Department of Crop Sciences, Tshwane University of Technology, Pretoria 0001, South Africa; zamandoccy24@gmail.com (N.P.C.); AkinolaSA@tut.ac.za (S.A.A.); ManhiviVE@tut.ac.za (V.E.M.); ShokoT@tut.ac.za (T.S.); 2 INRAE, Institut Agro Montpellier, SupAgro et, Université de Montpellier, F-34000 Montpellier, France; fabienne.remize@univ-reunion.fr; 3 Qualisud, Chemin de l’lrat, Université La Réunion, F-97410 Saint Pierre, France

**Keywords:** antioxidants, fermentation, indigenous mango cultivars, lactic acid bacteria strain, mango juice, volatile compounds

## Abstract

The effects of lactic acid fermentation using *Lactiplantibacillus plantarum* 75 (*L75*), *Leuconostoc pseudomesenteroides* 56 (*L56*) and its combination (*L56 + 75*) on the quality, bioactive and volatile compounds of mango juices (MJ) from three cultivars (‘Peach’, ‘Sabre’ and ‘Tommy Atkins’) were investigated. Fermented and unfermented MJ were evaluated for LAB growth, physicochemical parameters, volatile compounds, antioxidants activities (DPPH, ABTS, FRAP methods), total phenolic content (TPC) and sensory properties. The unfermented juices served as a control. Twenty-four-hour fermentation was ideal for MJ based on LAB growth profiles. Generally, titratable acidity, TPC, FRAP, DPPH and ABTS scavenging activities significantly increased with fermentation by the *L75* strain and were highest in the *L75*-fermented ‘Sabre’ MJ, while *L75*-fermented ‘Peach’ MJ had higher ABTS activity (*p* < 0.05). In contrast, the *L56* strain enhanced β-carotene retention, with improved colour properties in *L56*-fermented ‘Peach’ MJ. Fermentation with *L75* in ‘Sabre’ and ‘Peach’ MJ aided the synthesis of new volatile compounds (alcohols, esters, ketones and aldehydes). A PLS-DA scatter plot showed two clusters separating the ‘Peach’ and ‘Sabre’ mango juice fermented with *L75* from the rest. Based on the variable importance of the projection value (VIP) scores, pentadecane, 8-hexyl and butyl isobutyrate were shown as marker candidates to distinguish ‘Peach’ and ‘Sabre’ MJ fermented with *L75* from the other treatments, whereas ethyl octanoate and isobutyl acetate differentiated the ‘Sabre’ MJ fermented with *L75* from the other treatments. ‘Sabre’ and ‘Peach’ MJ fermented with *L75* and *L56* could provide antioxidants, meeting the recommended daily requirements for ascorbic acid and carotenoids in adults and teenagers. Hence, lactic acid fermentation of these local cultivars is a way to benefit consumers.

## 1. Introduction

Lactic acid bacteria (LAB) have been employed for decades in food fermentations. The fermentation process involves the use of carbohydrates to make organic acids, most notably lactic acid, and results in metabolites that have the potential to promote health [1]. In the last few decades, Lactobacilli have received increasing attention and some benefit of the recognition as probiotics that balance the gut microbiome [2]. Improved epithelial barrier integrity was reported in *Lactiplantibacillus plantarum* (*Ltp. plantarum*)-fermented tomato juice in an in vitro transepithelial electrical resistance assay [3]. Fermentation preserves foods but could also modify their organoleptic properties by producing a variety of flavours, aromas, textures and by eliminating antinutritive components [2]. Additionally, LAB fermentation of fruit juices is beneficial to vegans and lactose-intolerant consumers [3]. Fruit juices are well adapted to LAB fermentation due to their high sugar contents, nutrients (vitamin, mineral) and health-promoting compounds (fiber, phenolics and antioxidant) content [3]. According to Garcia et al. [4] LAB breaks down sugars in fruits and vegetables into acids, carbon dioxides and other flavour compounds, which helps modify the organoleptic properties of fermented foods, and, as an aside, improves their nutrient quality and bioaccessibility, safety and bioactivities.

Mango (*Mangifera indica*) fruit contains a range of antioxidants, including carotenoids, ascorbic acid, omega 3 and 6 polyunsaturated fatty acids, lutein, quercetin, mangiferin and flavonoids [5]. Mango fruit has a limited shelf life due to its highly perishable nature [6]. As a result, fermentation can be used to preserve this fruit [1]. *Ltp*. *plantarum* is the most widely used strain for lactic acid fermentation [7]. Several studies have suggested that fruit juices such as bergamot juices [8], pomegranate [9] and peaches [10] could serve as substrates for the fermentation and production of functional beverages. Furthermore, researchers have produced probiotic beverages using black cherry and barberry juices, watermelon and tomato juices using lactic acid bacteria as inoculum [11]. Another study reported the influence of brewer’s yeast and *Lactobacillus acidophilus* on the quality of beetroot juice and carrot juice [8]. It is possible that the differences in mango cultivars and LAB strains applied in fermentation could influence the physicochemical properties, soluble sugars, organic acids, volatile compounds and functional compounds in mango juice (MJ). Therefore, when developing functional juices, it is necessary to screen for the best cultivar that could produce fruit juice with optimum physicochemical, sensory and functional properties.

Our study tested the hypothesis that cultivar variation and different LAB strains do not affect the physicochemical properties, functional compounds, antioxidant properties, sensory properties or aroma compounds after fermentation. The mango cultivars studied were ‘Peach’ and ‘Sabre’, which are popular local mango varieties widely consumed by the rural communities in South Africa, and they were compared with the commercially available cultivar ‘Tommy Atkins’ mangoes. Physicochemical properties, functional compounds, antioxidant properties, sensory properties and aroma volatile compounds were determined at 24 h of fermentation after optimising the fermentation duration and were compared to the unfermented juice. It is anticipated that the results of this study will provide food manufacturers with information about the suitability of a specific cultivar to produce fermented MJ with good nutritional properties. It is the first study in which the LAB strains *Ltp. plantarum 75*, and *Leuconostoc pseudomesenteroides (Leu. pseudomesenteroides) 56* were tested for fermentation of local mango cultivars (‘peach’, ‘sabre’) juices singly or in combination and compared with the commercial export cultivar ‘Tommy Atkins’ mango juice.

## 2. Materials and Methods

### 2.1. Sample Preparation

The ripe ‘Peach’, ‘Sabre’ and ‘Tommy Atkins’ mango cultivars used in the study were purchased at an edible maturity stage from Tshakhuma market, Venda, Limpopo province, South Africa. All mango cultivars were disinfected by dipping in 1% (*v*/*v*) sodium hypochlorite solution, washed thoroughly in distilled water and peeled. The juice was extracted using a Russell Hobbs (700 W) household blender and diluted with sterile Valpre spring water (Coca-Cola, Cape Town, South Africa) at a ratio of 1:2 (*v*:*v*, juice:water). Mango juice (500 mL) was pasteurized at 85 °C in a water bath (WBE28A12E, PolyScience, Niles, IL 60714-4516, USA) for 15 min and cooled to room temperature (25 °C) before fermentation [9].

LAB strains used in the study were from the culture collections held by the University of La Réunion and QualiSud, France. *Ltp. plantarum 75* (*L75*) and *Leu. pseudomesenteroides 56* (*L56*) were isolated from cabbage [2]. ‘Peach’, ‘Sabre’ and ‘Tommy Atkins’ MJs were inoculated with LAB’s cultures as described by [10], with modifications. The LABs were reactivated and propagated for 48 h at 30 °C in 15 mL of de Man, Rogosa, and Sharpe (MRS) broth without agitation. The resulting cells were harvested by centrifugation at 1000× *g* for 10 min and suspended in sterile water, washed twice with sterile distilled water, and suspended in the same. The turbidity of LAB cultures was determined at 720 nm using a spectrophotometer (SPECTRO star Nano; BMG LABTECH, Ortenberg, Germany) and bacterial population was adjusted to 0.05 McFarland standard (5 × 10^8^ CFU/mL) [10]. Mango juice was inoculated with LABs’ cells at 1% of the total volume of juice and incubated at 30 °C for 72 h [11]. The juice was inoculated with both strains singly and in combination. The combined inoculum (*L56 + 75*) was developed at a ratio (1:1; *v*/*v*). Each fermentation was replicated five times, with unfermented juice as a control.

### 2.2. Microbial Analysis and Survival of Viable LAB Cells

The total counts of coliform, *Escherichia coli*, *Salmonella* spp., *Shigella* spp., mold and yeast and aerobic bacteria count were used to ascertain the safety of juices using the European commission regulation (EC 1441) [12]. Samples (1 mL) obtained from raw (0 h), un-inoculated stored at 4 °C (24 h), and fermented (24, 48 and 72 h) MJs were homogenised in 9 mL of sterile saline distilled water (0.85%), thereafter diluted serially to an appropriate population. (100 µL) of MJ were plated on appropriate media MRS agar, XLD (xylose lysine deoxycholate agar), MCA (MacConkey agar), PCA (plate count agar) and PDA (potato dextrose agar) in five replicates per sample. Inoculated plates were incubated aerobically at 37 °C for 24 h except PDA, at 25 °C for 48–72 h and MRS at 30 °C for 48 h. The viability of LAB was monitored at 24 h intervals during fermentation and expressed as Log CFU/mL of juice [10].

### 2.3. Evaluation of Sensory Attributes

Sensory analysis was determined using a qualitative descriptive analysis [13], with modifications. Nine healthy trained panellists, male and female between the age of 28 and 40 years were presented with fermented and unfermented MJs. Panellists were asked to identify desired attributes before test samples were presented to them for the assessment using an unstructured bipolar scale, after which it was extrapolated into intensity scores. References samples (Table S1) were presented to panellist during a three training sessions over a period of 2 weeks to identify attributes for evaluating fermented mango juice. Mango juices were presented in coded form to the panellists in white cups placed in a white light illuminated cubicle. The following sensory attributes; bright yellow colour, fruity aroma, mango flavour, consistency (viscosity), sweet taste, bitter taste, sour taste and overall drinking quality (acceptability) were evaluated after a consensus by the panellists on the desirable attributes in MJs. A commercial fermented product (unsweetened yoghurt), glucose syrup, ripe mango juice, quinine solution, sucrose solution and citric acid solution were used as references during the training sessions for the panellists. The attributes intensity scores were assigned based on absence (0), or presence of attribute; weak = 1, moderate = 5, and strong = 10. The panellists rinsed their mouths with water and ate cracker biscuits in between assessments of samples.

### 2.4. Determination of Physicochemical Parameters

To determine the titratable acidity, MJ was titrated with 0.1 N NaOH, 1%, and 3–4 drops of phenolphthalein indicator were used [14]. A refractometer (Agato pocket PAL-2, Tokyo, Japan) was used to measure the total soluble solids (TSS) of the fermented and unfermented MJs [15]. Following each test, the refractometer was calibrated with distilled water. A digital pH meter (Melter-Toledo Instruments Co., Shanghai, China) was used to measure the pH of unfermented and fermented MJs. The pH meter was calibrated using buffer solutions of pH 4.0 and 7.0.

The colour values of raw and fermented mango juice were measured using a colorimeter (CR-400 chroma Meter) calibrated with standard white tile [1] and measurements were made per sample of juice. As described in the CIE colour system, L* describes the lightness (white = 100, black = 0), a* refers to the intensity of redness (+) and greenness (−), b* refers to the intensity of yellowness (+) or blueness (−), and total colour differences (Δ*ε*) were calculated [10].

### 2.5. Ascorbic Acid Content

The ascorbic acid (Vitamin C) was determined using 2, 6-dichloroindophenol titration method [16]. The analysis was performed in five replicates and the values were expressed as mg of ascorbic acid per 100 mL of juice.

### 2.6. Total Phenol Content (TPC)

The Folin–Ciocalteu assay method with modifications was used to determine the juice’s TPC [10]. A 200-µL aliquot of a sample was mixed with 1000 µL of 10-fold diluted Folin–Ciocalteu reagent. A Na_2_CO_3_ solution (7.5%) was added to the sample and incubated for 2 h. The absorbance of the sample was measured at 750 nm using a spectrophotometer (SPECTROstar Nano; BMG LABTECH, Ortenberg, Germany). The blank was distilled water. The concentration was calculated using a calibration curve prepared with 1 µM gallic acid and the result was expressed in mg 100 mL^−1^ of juice.

### 2.7. β-Carotene Content

Briefly, an aliquot of mango juice (10 mL) was extracted using 5 mL of acetone: hexane (1:1) containing 0.1% (*w*/*v*) butylated hydroxytoluene (BHT) and kept in the dark overnight as described by Panfili et al. [17]. The mixture was centrifuged (Hermle Labortechnik, Germany Type 2326K, 2010, Wehingen, Germany) at 3420× *g* for 15 min at 25 °C. The residue was rinsed thrice with 5 mL of the extraction solvent and centrifuged. Afterwards, the supernatants were pooled, dried in anhydrous sodium sulphate, filtered through Whatman paper (No. 1) and evaporated under a stream of nitrogen until dry. Prior to analysis, the extracts were re-dissolved in 1 mL of isopropyl alcohol (10%) in n-hexane and filtered through a 0.45-m PTFE syringe filter. A Shimadzu Prominence-I LC-2030C 3D liquid chromatograph fitted with an LC-2030 autosampler and LC-2020/2040 PDA detector (Kyoto, Japan) was used for the characterisation and quantification of β-carotene. The column was a 250 mm × 4.6 mm id, 5-µm Shim pack GIST NH2 column. The wavelength was set at 460 nm at 30 °C and the injection volume was 10 µL with a flow rate of 0.6 mL min-1. β-carotene was identified by comparing retention times and absorption spectra with a pure standard. The standard curve was constructed from a range of pure β-carotene standard (0–100 g mL^−1^) at LOD (19.16) and LOQ (6.323) values and was used for quantification.

### 2.8. Antioxidant Activities

#### 2.8.1. 2,2-Diphenyl-1-picrylhydrazyl Radial Scavenging Activity

The antioxidants’ radical-scavenging activities of 2,2-diphenyl-1-picrylhydrazyl (DPPH) radicals were assayed. The DPPH solution in methanol (3.0 mL, 0.1 mM) was dispensed into 96-microplate wells along with various concentrations of the juice extract (0.5–15.0 mg in 150 mL methanol). After vigorous mixing, the samples were incubated for 30 min in the dark. Absorbance was measurements at 593 nm on a spectrophotometer (SPECTROstar Nano; BMG LABTECH GmbH, Ortenberg, Germany) [18]. The antioxidant concentration required to reduce DPPH absorbance by 50% was determined (IC_50_) and the results were expressed in mg mL^−1^ of juice.

#### 2.8.2. ABTS-Based Scavenging Activity

The ABTS radical cation decolorization assay was used to assess the free radical scavenging activity. The ABTS radical cation (ABTS^+^) was prepared by mixing ABTS stock solution (7 mM) with 4.9 mM potassium persulphate in a 1:1 ratio and the mixture was stored at 25 °C for 12–16 h [18]. In 1500 μL of ABTS^+^ solution, 40 μL of the sample (20, 80, 70, and 100 μM) was added. The mixture was incubated in a 96-well microplate reader at 37 °C for 10 min, under dark conditions. A spectrophotometer (SPECTROstar Nano; BMG LABTECH GmbH, Ortenberg, Germany) was used to measure the decrease in absorbance at 593 nm at a concentration of 40 μM of the sample (ABTS^+^). To calculate the IC_50_, the formula in Equation (1) was used, where: A = absorbance of control reaction; B = absorbance of the test sample. The results were expressed as mg mL^−1^.

(A − B) × 100/A (1)

#### 2.8.3. Antioxidant Power

A ferric reducing antioxidant power (FRAP) assay was conducted by combining TPTZ dissolved in 40 mM HCl with 20 mM of FeCl3 in a 1:1:10 ratio with a 20 mM acetate buffer (pH 3.6) [18]. The sample was homogenized in 80% methanol, and 20 µL of the sample and 150 µL of FRAP reagent were incubated for 10 min and the absorbance was measured at 593 nm using a microplate reader. Trolox solutions from 0 to 30 mM were prepared and used as a reference standard. Results were expressed as mM TEACg−1.

### 2.9. Volatile Compounds

Volatile constituents were extracted according to the method of Hijaz et al. [19] with a few modifications. Mango juice (10 mL) was placed in a 50-mm tube and 50 µL of internal standard butylated hydroxytoluene (1 mg/mL) and sodium chloride (1 g) was added. Volatile compounds was extracted using n-hexane (2 mL) and the solution was sonicated (MRC Ultrasonic cleaner, Model DC-150H, Cape Town, South Africa) at 30 °C for 30 min, and centrifuged (Type 2326K, 2010, Hermle Labortechnik, Wehingen, Germany) at 3420× *g* for 10 min and the supernatant was separated. The residue was rinsed thrice with n-hexane (2 mL) and centrifuged as above. The supernatants were pooled and dried with sodium sulphate, filtered with a white filter paper number 1, concentrated to 250 µL under a gentle stream of nitrogen, and, thereafter, stored at 5 °C before analysing using an Agilent 7890. A gas chromatograph (Agilent, Santa Clara, CA, USA) hyphenated to an Agilent 5975 C MSD with a triple-axis detector was used to analyse the volatile compounds present in non-fermented and fermented MJ. The machine was equipped with an auto sampler (Agilent Technologies GC Sampler 80). Helium was used as the carrier gas at a constant flow rate of 1 mL/min. Separation was done on a Zebron Guardian Capillary GC column (USA) with dimensions (30 mm × 0.25 mm i.d and 0.25-µm film thickness). The chromatographic oven program was as follows: 70 °C for 1 min then 3 °C/min to 142 °C for 0 min then 5 °C/min to 225 °C for 3 min, then 25 °C/min to 320 °C for 3 min; the total run time was 51.4 min. The split less injection was carried out at a 61.6 kpa pressure and 280 °C inlet temperature. The mass detector was operated with an electron energy of 70 eV in electron ionisation mode. The ion source and quadrupole temperatures were 230 °C and 150 °C, respectively, at an injection volume of 1 µL. Compounds were identified using the mass spectral library (NIST mass spectral library, Version 8). Alkanes were quantified using the pentadecane reference standard.

### 2.10. Statistical Analysis

Analysis was carried out in five replicates and the analysis of variance (ANOVA) was performed using statistical software (GenStat version 11.1, Hemel Hempstead, UK). Two-way ANOVA was employed to evaluate the effect of fermentation hour, LAB’s strains and cultivar and the means were separated using Fischer’s least significant difference at 95% significance. Results were presented as mean ± SD. MetaboAnalyst was used to establish the correlation pattern of juice volatile compounds using the PCA, PLS-DA, heat map and clustering analysis.

## 3. Results and Discussion

### 3.1. Total Soluble Solids (TSS)

TSS is largely responsible for determining the sweetness of fruit juices. The TSS of fresh ‘Peach’ MJ (3.42 °Brix) was higher than un-inoculated ‘Sabre’ (2.40 °Brix) and ‘Tommy Atkins’ (1.76 °Brix) juices (Table 1). Fermentation of ‘Peach’ or ‘Tommy Atkins’ MJs with *L75*, *L56*, or *L56 + 75* decreased the TSS compared with their unfermented counterparts. Meanwhile, ‘Sabre’ MJ fermented with *L75* was significantly different from the unfermented juices (at 0 and 2 h) in terms of TSS, while it was not the case in *L56* and *L56 + 75*-fermented juices. The ‘Peach’ MJ fermented with *L75* had the lowest TSS content, which represented a 50% decrease of TSS due to fermentation. Our observations agree with the decline in TSS of mango juice and sapota juice fermented with *Ltp. plantarum.* NCDC LP 20 for 24 h, 48 h and 72 h [13]. Additionally, the sugar depletion in *Ltp. plantarum* fermented cashew apple juice proceeded at a much faster rate than other LAB’s during the fermentation process [20], similar to our observation. The decreasing TSS could be of health benefit to diabetic and obese patients, as it could limit the excessive intake of sugars that are abundant in juices.

### 3.2. pH and Titratable Acidity

Overall, fermentation of juices from ‘Peach’, ‘Sabre’ and ‘Tommy Atkins’ mango cultivars with different LAB strains in combination or stand-alone resulted in a reduction in pH compared with their corresponding unfermented juices at 0 or 24 h (Table 1). The pH decreased following the pattern (*L75* > *L56 + 75* > *L56*) in all cultivars, ranging from 3.66 to 4.85 and was lowest in *L75*-fermented ‘Sabre’ MJ (3.66). According to Mashitoa et al. [21] the pH of mango puree fermented with *Leu. pseudomesenteroides* 56 and *Ltp. plantarum* 75 decreased after fermentation. In the same way, *Leu. mesenteroides* MPL18 and MPL39 decreased the pH of mango juice after fermentation [22]. The degree of lactic acid production is dependent on LAB’s strains, and it could be the reason for the observed variation in pH in this study [1].

Consistently, fermentation led to an increase in titratable acidity (TA) in ‘Peach’ and ‘Sabre’ MJs fermented with *L75*, *L56* and *L56 + 75* compared with the unfermented juices, except the ‘Tommy Atkins’ cultivar (Table 1). The TA in fermented MJs ‘Peach’, ‘Tommy Atkins’ and ‘Sabre’ were in the ranges of 27–30, 26–28 and 35–36 g/100 g lactic acid, respectively. However, *L75*-fermented juices had a significant higher TA compared to other LABs’ fermented and unfermented mango cultivar juices (*p* ≤ 0.05). On the other hand, *L56* or *L56 + 75*-fermented ‘Tommy Atkins’ juice showed no significant changes in the TA (*p* ≥ 0.05). Higher TA was observed in fermented ‘Sabre’ mango juice than in ‘Peach’ and ‘Tommy Atkins’ mango juices. A similar observation of increased TA was reported in sapota juice fermented with *Ltp. plantarum* NCDC LP 20 for 24 h [9]. Therefore, a fall in pH is a result of the fermentation process, where LAB metabolizes simple sugars such as sucrose, fructose, and glucose into organic acids, mainly lactic acid and carbon dioxide [2].

### 3.3. Colour Properties

Indicators of quality include colour intensity. Luminosity, brightness, colour coordinate (b*), and chroma (c*) of the yellow colour ‘Peach’ mango juice fermented by *L75* or *L56* were similar to that when unfermented at 0 h (Table 2), suggesting that there was no colour change in juice during fermentation. Similarly, the colour change (Δ*ε*) value was lower with LAB fermentation for 24 h compared with the unfermented condition. The colour difference was lowest in *L56*-fermented juices across mango cultivars and, furthermore, it was lowest in ‘Peach’ MJ (1.41). This observation could be due to increased acidity of the fermented juices which help protect against enzymatic browning. Food products with dark colours are less likely to be accepted by consumers and colour change equal to or greater than two is considered a significant colour difference in samples [10].

The observed colour change in un-inoculated (U 24 h) mango juices from all cultivars could be due to an enzymatic browning activity [21]. However, the production of alcohols and organic acids due to the hydrolysis of free sugars by the LAB’s could have possibly prevented the enzymatic browning [23] in ‘Peach’, ‘Sabre’ and ‘Tommy Atkins’ mango juices. In the combined culture (*L56 + 75)*, significant reductions in colour attributes (L*, a* and *chroma*) were observed in ‘Peach’ and ‘Sabre’ MJs relative to the *L56* and *L75*-fermented juices, possibly due to synergistic oxidative activities of the homo-fermentative LAB’s (*L75* or *L56*) causing the fast triggering of an enzymatic colour degradation process. In ‘Tommy Atkins’ juice, the *L56 + 75* had a non-significant decline in colour properties compared with *L75* and *L56* juices but significantly different from the unfermented juices (*p* > 0.05).

### 3.4. Microbial Quality and LAB Growth

**Figure 1:**
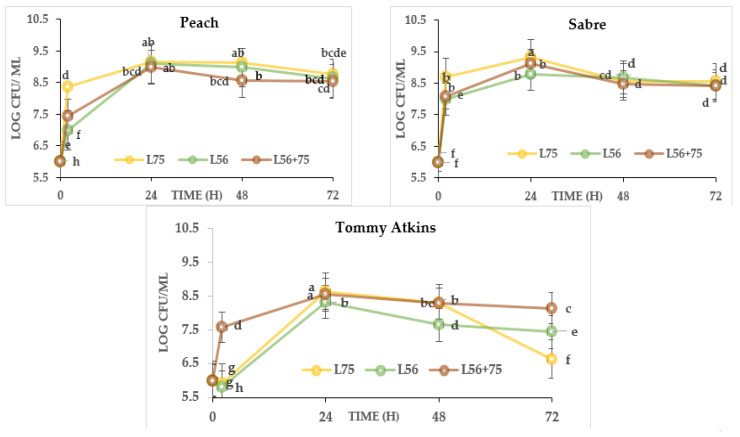
Population of lactic acid bacteria in fermented mango juice from different cultivars. Alphabets indicates significant difference at *p* ≤ 0.05. Keys: *Ltp. plantarum (L75); Leu. pseudomesenteroides (L56); Leu. pseudomesenteroides 56 + Ltp. plantarum 75 (L56 + 75)*.

The absence of growth in fermented and unfermented juices plated on XLD, MCA, PCA and PDA media (Table S2A–C) is an indication that there was no contamination in the MJs from different cultivars, therefore these samples meet the public health criteria (<10 cfu/100 mL of juice) for microbial safe juice [22]. No *Salmonella*, *E. coli*, coliforms or *Shigella* spp. were detected in the fermented or unfermented MJ from the selected cultivars. The total fungi count in fermented and unfermented MJ were in the range; 0.02–4.43, 0.02–0.43 and 0.01–3.46 Log CFU/mL, while the total aerobic bacterial count ranged from 1.6–9.33, 2.56–9.83 and 0.63–8.4 Log CFU/mL in ‘Peach’, ‘Sabre’ and ‘Tommy Atkins’ MJs, respectively. The yeast and total aerobic bacterial count was highest in *L75*, 24-h-fermented juices across all cultivars. With respect to cultivars, ‘Tommy Atkins’ had the lowest aerobic bacteria count while ‘Sabre’ had the highest bacterial and yeast counts in fermented MJ. According to Managa et al. [10], LAB are capable of co-existing in a consortium with acid tolerant fungi (yeast) during fermentation. A significant decrease in yeast and total aerobic bacterial count was observed in fermented MJs across cultivars (*p* ≤ 0.05), while no growth was obtained in the non-fermented samples, thus signifying a contamination-free and safe production process. No LAB growth was observed in the unfermented juice across the mango cultivars (Figure 1). After 2 h of fermentation, LABs had a significant increase in population and were greatest in *L75* samples relative to the *L56* and combined cultures *(L56 + 75)* in all mango juices. This corresponded to the beginning of the growth phase for the LAB cultures, while, at 24 h, they ended the logarithmic growth phase. At 24 h, the LAB’s count ranged from 8.99 to 9.17 log CFU/mL in ‘Peach’, ‘Sabre’ (8.79–9.31 log CFU/mL) and ‘Tommy Atkins’ (8.31–8.62 log CFU/mL). The highest population was observed in *L75*-fermented juices (‘Sabre’: 9.31 log CFU/mL; ‘Peach’: 9.17 log CFU/mL and ‘Tommy Atkins’: 8.62 log CFU/mL) compared with *L56*- and *L56 + 75*-fermented MJs from different cultivars.

However, a decline in LAB population at 48 h before reaching a stationary phase (*p* ≤ 0.05) was observed in all cultivars with the smallest decrease in ‘Peach’ fermented with *L75* (0.5%). L56 + 75 reached the stationary phase with a 4.78% decline at 48 h (8.56 Log CFU/mL) and thereafter maintained stability at 72 h. A significant decline in the LAB population of *L56* + 75-fermented MJs from all cultivars could be due to LAB’s competing for the potential use-up of nutrients and the build-up of metabolites which could constitute a toxic system. Probably the lower pH and increased acidity could have caused the slight reduction in the survival of LAB strains at 48 h. Acids inhibit bacterial growth and viability by acidifying the cytoplasm, increasing energy consumption necessary for the maintenance of intracellular pH, and inhibiting enzyme reactions [24]. A lower LAB population was observed in the ‘Tommy Atkins’ juices relative to other cultivars. However, significantly higher counts in all cultivar juices fermented with *L75* could be due to the versatile nature of *Ltp. plantarum* 75 in fermentation [24], while the lower sugar content in ‘Tommy Atkins’ MJ [25] could have made fermentation proceed slowly. Therefore, 24-h fermentation was selected and used in this study.

### 3.5. Sensory Analysis

**Figure 2:**
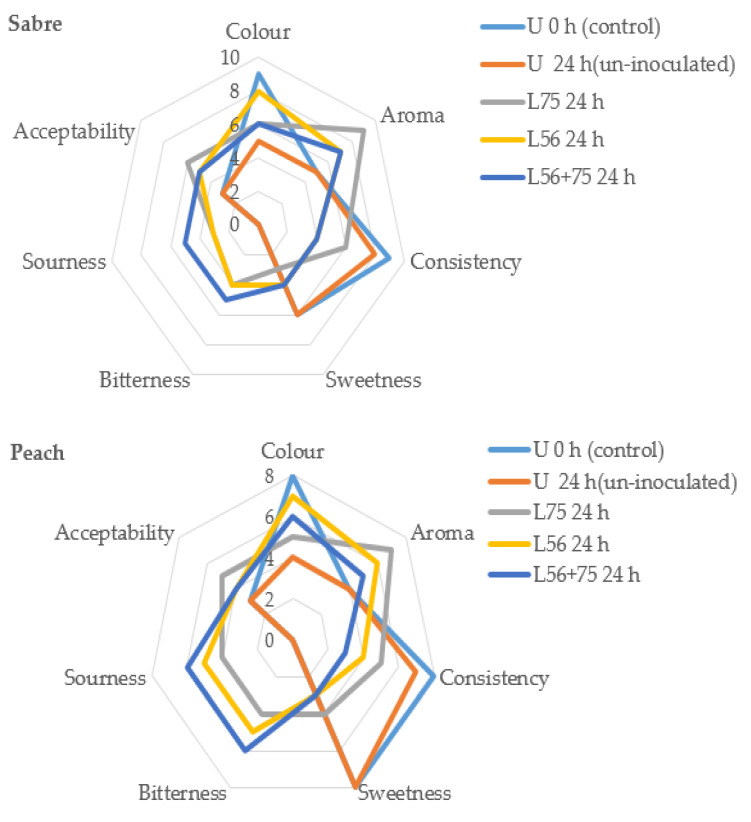

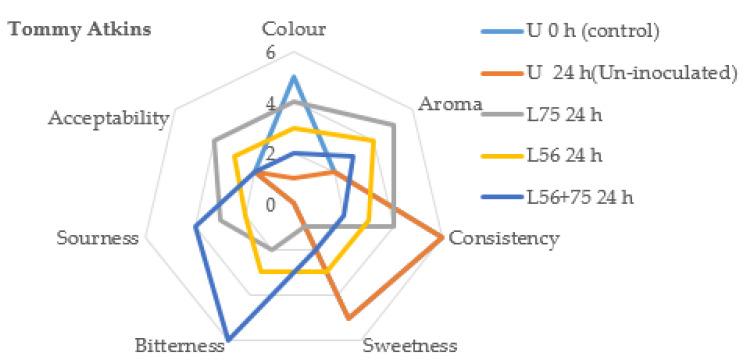
Sensory attributes in juice from different mango cultivars fermented by different lactic acid bacteria. Keys: U 0 h—raw unfermented mango juice (control); U 24 h—un-inoculated mango juice stored for 24 h; *Ltp. plantarum* (*L75*)*; Leu. psuedomesenteroides* (*L56*); *Leu. psuedomesenteroides56 + Ltp. plantarum 75* (*L56 + 75*).

The sensory attributes of LAB fermented and unfermented mango juices from ‘Peach’, ‘Sabre’ and ‘Tommy Atkins’ cultivars are presented in Figure 2. The bright yellow colour perception in mango cultivar juices was influenced by cultivar type and duration of fermentation. In ‘Sabre’ mango cultivar, colour perception ranged from slight yellow colour (5) in un-inoculated MJ (U 24 h) to bright yellow colour (9) in fresh juice (U 0 h), while in ‘Tommy Atkins’ juices, colour perception ranged from dark yellow colour (2) in *L75*- and *L56 + 75*-fermented juices to slight yellow colour in *L56*-fermented juice. The colour perception of the ‘Peach’ mango cultivar ranged from weak yellow colour (4) to bright yellow (8) in the fresh juice. Fermentation and storage caused a decrease in the bright yellow colour of MJs from different cultivars, however, the *L56* strain aided the preservation of the yellow colour in fermented mango cultivar juices, which probably related to the inhibition of enzymatic colour change and preservation of the carotenoids. The decrease in the yellow colour perception could be due to an autoxidation process of the poly-phenolics in the juice thereby causing browning [25]. *L75* strain increased the aroma perception in fermented mango juices of all cultivars. The highest aroma perception was reported by the panellists in ‘Sabre’ MJ fermented with *L75*. Consistency and sweetness were reduced in fermented mango juices of all cultivars, while bitter and sour taste increased in fermented juices. The degree of sourness was highest in ‘Peach’ MJ fermented with *L56 + 75,* while a higher bitter taste was observed in ‘Tommy Atkins’ and ‘Peach’ MJs fermented with *L56 + 75*. The lower sour taste in ‘Tommy Atkins’ MJ could be due to the lower sugar content in this cultivar [26] thereby resulting in reduced fermentation. Panellists accepted the ‘Sabre’ MJ fermented with *L75* and sensory quality was comparable to the commercial juice. The higher acceptability of ‘Sabre’-fermented juices by the panellists could be due to their mild acid taste compared to ‘Peach’ MJs.

### 3.6. Chemical Constituents

#### 3.6.1. Ascorbic Acid

The ‘Sabre’ fresh mango juice had the highest ascorbic acid (AA) content (15 mg 100 mL^−1^) and *L75*-fermented ‘Sabre’ mango juice had the highest AA retention (11.23 mg/100 g) of the three mango cultivars (Table 3). However, the AA content decreased significantly at 24 h of fermentation. It could be that the ascorbic acid oxidase in mango juices was not completely inactivated by pasteurization, resulting in its degradation [20]. Generally, *L75*-fermented mango juices had the lowest reduction in AA during fermentation. A similar decrease in AA was reported in Washington navel orange juice fermented with *Ltp. plantarum* [27]. The observed differences in AA concentrations of unfermented juices could be attributed to the differences in cultivar types and pH values (Table 1). Acidic pH inhibits the autoxidation of AA when the redox potential changes [28]. According to this study, the effect of fermentation on AA is matrix and LAB strain specific, which could have contributed to the greater metabolism of *L75* and its lower pH level [1,27]. Women and men require 75 and 90 mg of AA daily, respectively [29]. It is noteworthy that 2.67 and 3.2 glass (250 mL) of ‘sabre’ MJ fermented with *L75* could satisfy this daily requirement in adult women and men, respectively.

#### 3.6.2. Total Phenol Content

The TPC in un-inoculated MJs stored for 24 h decreased in the juices of ‘Peach’ and ‘Sabre’ mango cultivars, and the greatest decrease was obtained in ‘Sabre’ (13.7%) (Table 3). This observation could be the result of enzymatic oxidation that advances the oxidation of phenolics to quinones [30]. A similar decrease in TPC was reported in strawberries [31] stored for 24 h. Contrarily, the un-inoculated ‘Tommy Atkins’ juice had a significant increase in TPC (18.05%), probably due to depolymerisation of complex glucosides in the juice. The three mango cultivar juices fermented with *L75* and *L56* showed an increase in TPC, with the greatest increase in ‘Sabre’ juice fermented with *L75* after 24 h (1415.71 mg/100 mL). In contrast, *L56 + 75* increased the TPC of ‘Peach’, and ‘Sabre’ MJs by 16.48% and 3.79%, respectively, while a 10.8% decrease was observed in ‘Tommy Atkins’ juice. The decrease in *L56 + 75*-fermented ‘Tommy Atkins’ juice could be due to a low sugar matrix in the juice and lower concentration of the versatile fermentative *L75* strains used relative to *L75* singly inoculated juices.

LAB biotransformation of phenolics is matrix- and LAB-strain-dependent in mango juices, thus supporting the findings of [27] in fermented Washington navel and ‘Tarocco’ orange juices. Thus juices from mango cultivars (‘Peach’, ‘Sabre’ and ‘Tommy Atkins’) may have exhibited unique chemical characteristics that may have either facilitated or reduced the growth and enzymatic activities of LAB strains and, consequently, the amount of TPC conversion in the juices. According to Landete et al. [32] glycosylated phenolics in mulberry juice are deglycosylated by *Ltp. plantarum* during fermentation, thus releasing soluble conjugated or insoluble bound phenolic compounds from plant cell walls. Further, the differences in the TPC concentrations among the LAB strains-fermented MJs suggest the ability of the strains to produce more hydrolytic enzymes is highly dependent on the matrix.

#### 3.6.3. β-Carotene

The β-carotene content in all three mango juices increased significantly after fermentation with *L75*, or *L56* or *L56 + 75* when compared to the unfermented juices at 0 or 24 h (Table 3). However, fermentation with *L56* consistently increased the β-carotene content in all the mango cultivars juices, and the highest concentration was obtained in ‘Peach’ juice (19.89 µg mL^−1^) and lowest in ‘Tommy Atkins’ (0.15 µg mL^−1^). As suggested by Venil et al. [33] the increase in β carotene during lactic acid bacteria fermentation could be due to an increase in the macromolecular changes that serves as a protective mechanism against oxidative stress and improves the extractability of carotenoids due to its enzymatic activity. The lower β-carotene content in *L75* relative to *L56*-fermented juices from all cultivars supports the report of slight carotenoid degradation in *Ltp. plantarum*- and *Lb. fermentum*-fermented carrot juice [34]. Lactic acid bacteria fermentation can enhance the bioavailability and release of phytochemicals in the plant matrix, such as carotenoids [34]. It is possible that the use of a combined starter culture could have increased the endogenous lipoxygenase activity and degraded the β-carotene content in juices [35]. As 9–13-year-old boys and girls requires 600 mcg RAE of β-carotene daily, hence, 1.21 glass of 250 mL of *L56* fermented ‘Peach’ mango could deliver the daily requirement of β-carotene for teenagers.

### 3.7. Antioxidant Activity

As shown in Table 3, there was a significant increase in antioxidant power in all fermented mango juices from the three cultivars after fermentation. FRAP was significantly high in *L75* fermented juices across cultivars and was highest in *L75*-fermented ‘Sabre’ mango juice (558.28 µM TEAC/mL) compared with the unfermented and un-inoculated juices (*p* < 0.05). In contrast, *L56*-strain-fermented ‘Peach’ and ‘Sabre’ mango juices and *L56 + 75*-fermented ‘Tommy Atkins’ MJ had significantly lower FRAP activities relative to the fermented juices. The positive influence of *L75* on the antioxidant activity of the ‘Peach’ and ‘Sabre’ mango juices could be due to their hydrolytic capacity to liberate active compounds during fermentation. In addition, the antioxidant scavenging activities (DPPH) were greater in all three mango cultivar juices fermented with *L75* compared with other fermented juices. The highest IC50 DPPH activity was in *L75*-fermented ‘Sabre’ juice (44.7 mg/mL), while *L75*-fermented ‘Tommy Atkins’ cultivar juice was not significantly different from the control (*p* > 0.05). The antioxidant ascorbic acid is a free radical scavenger and it can scavenge DPPH radicals [36], thus, corroborating the higher AA content in *L75*-fermented ‘Sabre’ juice. Additionally, lowering the pH increases the stability of ascorbic acid and slows its transformation to dehydroascorbic acid, which supports DPPH scavenging [37]. Moreover, its reaction is very fast as compared with that of other molecules, such as polyphenols, that can scavenge free radicals [25]. ABTS radical-scavenging activities are frequently used methods to evaluate antioxidant activity; free radicals become stable when they accept hydrogen ions from antioxidants, so their blue colour is lost [38]. ABTS radical-scavenging activity increased in the three mango cultivars fermented with *L75*, with ‘Peach’ mango juice showing the highest IC_50_ level (14.51 mg/mL).

### 3.8. Effects of Fermentation Process on Volatile Compounds

**Figure 3:**
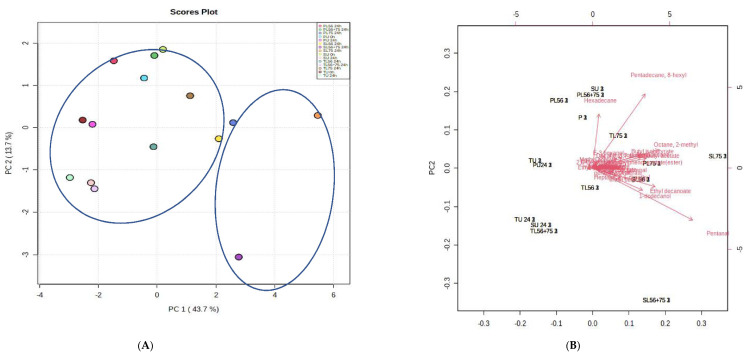

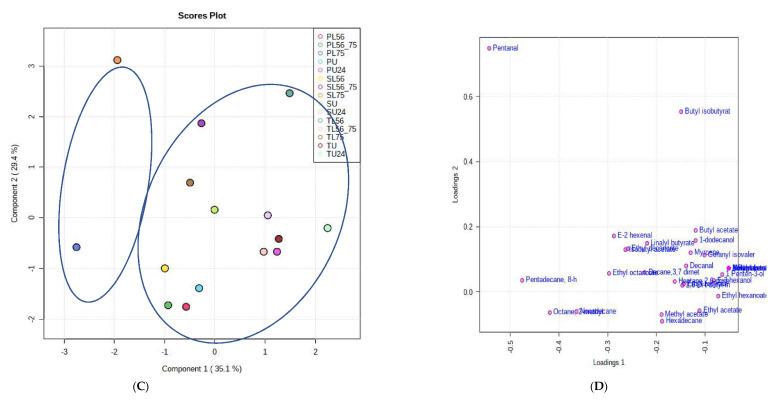

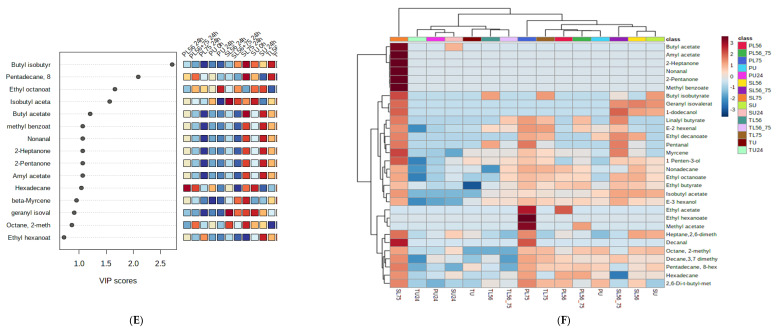
(**A**). PCA score plot and, of volatile compounds in fermented mango juices. (**B**). PCA loadings of volatile compounds against antioxidants and antioxidant activities of fermented mango juices from different cultivars. (**C**) PLS-DA score plot (R2Y = 0.66, R2X = 0.70, and Q2 = 0.51) (**D**). PLS-DA loadings, (**E**) VIP results of volatile compounds of fermented mango juices. (**F**) Heat map and hierarchical clustering of volatile compounds in mango juice before and after fermentation. Keys: U 0 h-raw unfermented mango juice; U 24 h-un-inoculated mango juice stored for 24 h; *L75: Lactiplantibacillus plantarum* 75; *Leuconostoc pseudomesenteroides 56* (*L56*); *Leuconostoc pseudomesenteroides 56 + Lactiplantibacillus plantarum 75* (*L56 + 75*); P-‘Peach’; S-‘Sabre’; T-‘Tommy Atkins’ cultivars.

A total of 171.71, 169.19 and 84.14 µg/mL of volatile compounds were found in the raw juice of ‘Peach’, ‘Sabre’ and ‘Tommy Atkins’ mango cultivars, respectively (Table 4). The total volatile compounds decreased to 76.23, 96.3 and 67.84 µg/mL in unfermented mango cultivars (‘Peach’, ‘Sabre’ and ‘Tommy Atkins’) juices, respectively, after 24 h of fermentation (Table 4). Fermentation with the *L75* strain increased the total volatile compounds in MJs from different cultivars compared with *L56* and *L56 + 75* juices. ‘Sabre’ mango juice fermented with *L75* after 24 h had the highest volatile compounds content (27) relative to other juices, comprising alkanes (6), ketones (2), esters (14), aldehydes (4), alcohols (3) and a terpene hydrocarbon. In order to identify potential discriminant variables, multivariate data analyses, such as principal component analysis (PCA) and partial least squares-discriminant analysis (PLS-DA) were employed to reduce the complexity of the data set, as shown in Figure 3A–D. Score plots of PCA for the data set of volatile compounds are shown in Figure 3A. Based on PCA analysis of all volatile compounds, two principal components (PC) explained 57.4% of the overall variance in aroma compounds during fermentation (Figure 3B). PC1 explained 43.7% of the variance indicating that ‘Peach’ and ‘Sabre’ mango juices fermented with *L75* and ‘Sabre’ mango juice fermented with the combined starter (*L56 + 75)* contained high amounts of pentanal. Pentanal correlated positively on PC1 and separated the ‘Peach’, and ‘Sabre’ mango juices fermented with *L75* and *L56 + 75* from other treatments.

PLS-DA scatter plot showed two clusters separating the ‘Peach’ and ‘Sabre’ MJ fermented with *L75* from the rest (Figure 3B). Analyses of the PLS-DA results showed that the samples tested were classified differently. Variable importance in the projection value (VIP) of the marker compounds exceeded 1.0 (Figure 3C), and the 11 marker compounds were hexadecane (1.3), amyl acetate (1.3), 2-pentanone (1.31), 2-heptanone (1.3), nonanal (1.32), methyl benzoate (1.30), butyl acetate (1.52), isobutyl acetate (1.6), ethyl octanoate (1.72); pentadecane-8-hexyl (2.13) and butyl isobutyrate (2.70). A variable’s VIP score is calculated as a weighted sum of the squared correlations between the PLS-DA components and the original variable. PLS-DA in the model explains the variation in percentage terms (weights). Moreover, the volatile compounds butyl acetate (1.52), isobutyl acetate (1.6), ethyl octanoate (1.72); pentadecane-8-hexyl (2.13), butyl isobutyrate (2.70) demonstrated a VIP score > 1.5. Based on the VIP scores, pentadecane- 8-hexyl and butyl isobutyrate could be used as markers to distinguish ‘Peach’ and ‘Sabre’ mango juices fermented with *L75* from the rest of the treatments. Ethyl octanoate and isobutyl acetate separated the ‘Sabre’ mango juice fermented with *L75* from the other treatments.

The ‘Sabre’ MJ fermented with *L75* produced more volatile compounds than did other treatments (Table 4). The main volatile compounds generated during fermentation of ‘Sabre’ MJ by *L75* were pentadecane, 8-hexyl (60.9 µg/mL) ethyl octanoate (30.60 µg/mL), decane-3,7-dimethyl (26.2 µg/mL), butyl acetate (22.6 µg/mL), 2,6-di-t-butylmethylphenol acetate (84.9 µg/mL) ethyl butyrate (49.02 µg/mL) and penten-3-ol (36.7 µg/mL), as shown in Table 4. Using hierarchical cluster analysis, the relationship between volatile compounds and mango juice fermented with LAB cultures was uncovered.

As shown in 3D, the mango juices fermented with different LAB cultures were grouped into two clusters in the heat-map. The first cluster was found in ‘Sabre’ MJ fermented by *L75* with a higher proportion of butyl acetate, amyl acetate, 2-heptanone, nonanal, 2-pentanone, and methyl benzoate. The second cluster included all other cultivars such as ‘Peach’ or ‘Tommy Atkins’ mango juices fermented with different LAB cultures and controls. Results suggest that fermentation of ‘Sabre’ mango juices with *L75* significantly improved aroma volatile compound formation. Additionally, ethyl acetate, ethyl hexanoate and methyl acetate were only detected in ‘Peach’ MJ fermented with *L75* at higher concentrations compared with other treatments and was not found in ‘Sabre’ MJ fermented with *L75*.

Esters positively influence the fruity flavour of the juices and enhance the sensory profile [39]. In unfermented raw ‘Peach’ ‘Sabre’, and ‘Tommy Atkins’ MJs, alcohols accounted for 23.65%, 28.19% and 52.08% of total volatile compounds, respectively. However, the alcohol recovered in the fermented MJs varied from 14.73 to 24.54% (Peach), 14.69 to 26.41% (Sabre) and 21.25% to 33.08% (Tommy Atkins). Possibly, the alcohols were used to produce esters during fermentation. Ester synthesis is largely dependent on alcohol production since fatty acyl-CoA is the major ester formed during the synthesis pathway in lactic acid fermentation [1]. Similarly, the synthesis of esters is dependent on the occurrence and activity levels of esterase [40]. Activities of the alcohol acyltransferase enzyme are involved in ester biosynthesis [41]. However, the by-products of ethanol fermentation (ethyl acetate), was found only in ‘Peach’ juice fermented with *L75* in this investigation. The major contributor to the green taste was (E)-3 hexanol, which was found in higher concentration in ‘Peach’ (24.46%), ‘Sabre’ (36.79%) and ‘Tommy Atkins’ (31.55%) MJs fermented by *L75* and in ‘Sabre’ MJ fermented by *L56 + 75* (36.68%) compared with their raw counterpart. Equally, the percentage increase in 1-penten-3-ol (fruity, and green aroma) was highest in *L75*-fermented ‘Sabre’ MJ (80.23%) followed by ‘Peach’ MJ (74.99%) and was significantly different from other juices relative to the raw juice (U 0 h).

Differences in the genetic make-up of the strains, metabolism type and the type of available sugars (carbohydrate) in substrate could be the reason for the observed differences in aroma volatile compounds observed with these different LAB strains [40]. Nonanal (rose-orange flavour) was a newly produced aldehyde in ‘sabre’ MJ fermented with *L75.* Nonanal was not detected in ‘Sabre’ MJ fermented by *L56, L56 + 75*, nor in fermented ‘Peach’ and ‘Tommy Atkins’ mango juices. ‘Peach’ and ‘Sabre’ MJ fermented with *L75* had the highest levels of newly formed pentanal (fruity note), followed by ‘Sabre’ MJ fermented with *L56 + 75*. Newly formed volatile (decanal) was detected only in ‘Peach’ (1.71 µg/mL) and ‘Sabre’ (2.70 µg/mL) mango juices fermented by *L75*. *L75* fermented ‘Peach’ and ‘Sabre’ MJs had a higher E-2 hexanal (green note) than *L56*, *L56 + 75* or ‘Tommy Atkins’ MJ fermented with any of the three LAB strains. Aldehydes are typically unstable compounds that are either reduced to alcohol or oxidized to acids in food matrices [39]. Diverse enzymes in lactic acid bacteria reduces aldehydes into alcohols or oxidize them into acids. This might explain why some aldehydes and alcohols were absent at lower levels in *L56* and *L56 + 75*-fermented mango juices. ‘Sabre’ MJ fermented with *L75* was the most abundant in ketones (2-pentanone and 2-heptanone), which were not detected in the unfermented nor other fermented juices of ‘Peach’ and ‘Tommy Atkins’ cultivars. All alkanes (octane-2-methyl, decane-3,7-dimethyl, nonadecane, hexadecane, pentadecane 8-hexyl, heptane-2,6-dimethyl) increased significantly in ‘Sabre’ mango juices fermented with *L75* (*p* ≤ 0.05).

Furthermore, ‘Sabre’ MJ fermented with *L75* produced higher levels of β-myrcene compared to other fermented and raw mango cultivar juices. A similar increase in β-myrcene was reported in *Ltp. plantarum* POM1- and LP09-fermented pomegranate juices [42]. Monoterpenes in MJ are either free or glycosidically conjugated [41]. Due to the low pH of the juices, lactic acid fermentation or enzymatic hydrolysis (β-glucosidase) may account for the highest (*p* ≤ 0.05) levels of terpene (β-myrcene) found in ‘Sabre’, ‘Peach’, and ‘Tommy Atkins’ mango juices. Acid hydrolysis changes the monoterpene aglycones and dramatically reduces their aroma, but enzymatic hydrolysis does not alter the monoterpene aglycones, and it appears to be a better method of liberating volatile monoterpenes [42]. Several desirable alcohols, aldehydes, ketones, terpenes and esters are apparent in the ‘Sabre’ mango juice fermented with *L75* (green, floral, and fruity notes).

## 4. Conclusions

Fermentation of ‘Sabre’, ‘Peach’ and ‘Tommy Atkins’ mango juices improves the quality its functionality. Lactic acid fermentation of mango cultivar juices is dependent on the type of strain causing the fermentation, mango cultivar and duration of fermentation. From this study, fermentation by *L75*, *L56* and *L56 + 75* for 24 h in mango cultivar (‘Sabre’, ‘Peach’ and ‘Tommy Atkins’) juices was considered suitable for quality mango juice. *L75*-fermented ‘Sabre’ mango juice showed the potential to function as a probiotic due to its higher LAB survival. The *L56* strain helped to retain the carotenoid content in the juices of mango cultivars and influenced the yellow colour perception. Approximately a glass of *L56* fermented ‘Peach’ mango juice could provide the daily requirement of β-carotene for teenagers. Therefore, frequent intake of ‘Peach’ mango juice fermented by *L56* could be an ideal strategy to manage cases of night blindness in rural communities. Furthermore, the *L75* strain improved the physiochemical, volatile compounds, ascorbic acid, phenolic content and antioxidants activities of fermented mango cultivar juices. Fermentation of ‘Sabre’ mango juice by *L75* aids the synthesis of new alcohols, esters, ketones and aldehydes. Due to the improved physicochemical and bioactive properties, *L75* fermented ‘Sabre’ mango juice is considered most preferred and able to deliver a functional benefit to health. The lower pH and TSS contents in *L75*-fermented juices made them suitable for people with diabetic conditions, thereby aiding the limiting of sugar intake. However, further investigations on the organic acids produced during the fermentation process should be further investigated. Hence, the fermentation of local mango cultivar ‘Sabre’ with *L75* strain is acceptable and could be recommended to local and food processors in South Africa for better quality mango juice production.

## Figures and Tables

**Table 1:** Changes in physicochemical parameters of mango juices as influenced by different lactic acid bacteria.

Mango Cultivar Juice	TSS (°Brix)	pH	TA (Equivalent Lactic Acid g/100 g)
‘peach’ mango juice			
U 0 h (Raw)	3.42 ± 0.27 ^a^	4.84 ± 0.03 ^a^	20.00 ± 0.02 ^e^
U 24 h (un-inoculated)	3.42 ± 0.29 ^a^	4.85 ± 0.05 ^a^	21.00 ± 0.01 ^e^
*L75* 24 h	1.72 ± 0.08 ^d^	3.88 ± 0.01 ^b,c^	30.00 ± 0.01 ^b,c^
*L56* 24 h	2.14 ± 0.17 ^b^	3.96 ± 0.01 ^b^	27.00 ± 0.03 ^c,d^
*L56 + 75* 24 h	2.03 ± 0.04 ^c^	3.89 ± 0.01 ^b,c^	29.00 ± 0.01 ^c^
‘sabre’ mango juice			
U 0 h (Raw)	2.40 ± 0.07 ^b^	4.28 ± 0.36 ^a^	23.00 ± 0.03 ^d,e^
U 24 h (un-inoculated)	2.30 ± 0.07 ^b^	4.18 ± 0.11 ^a,b^	23.00 ± 0.02 ^d,e^
*L75* 24 h	1.52 ± 0.08 ^d^	3.66 ± 0.03 ^d^	36.00 ± 0.04 ^a^
*L56* 24 h	2.24 ± 0.11 ^b^	3.88 ± 0.01 ^c^	35.00 ± 0.04 ^a,b^
*L56 + 75* 24 h	2.26 ± 0.11 ^b^	3.80 ± 0.05 ^c^	36.00 ± 0.03 ^a^
‘Tommy Atkins’ mango juice			
U 0 h (Raw)	1.76 ± 0.05 ^d^	4.12 ± 0.01 ^a^	23.00 ± 0.05 ^d,e^
U 24 h (un-inoculated)	1.76 ± 0.05 ^d^	4.12 ± 0.01 ^a^	24.00 ± 0.02 ^d,e^
*L75* 24 h	0.62 ± 0.04 ^e^	4.01 ± 0.01 ^c^	28.00 ± 0.02 ^c^
*L56* 24 h	0.72 ± 0.08 ^e^	4.07 ± 0.04 ^b^	26.00 ± 0.02 ^c,d^
*L56 + 75* 24 h	0.70 ± 0.07 ^e^	4.03 ± 0.01 ^c^	27.00 ± 0.04 ^c,d^
LSD	2.44 *	0.01 **	0.04 ***

Values with the same alphabetic letter along the column are not significantly different (*p* ≤ 0.05). Keys: U 0 h—raw unfermented mango juice (control); U 24 h—un-inoculated mango juice stored for 24 h; *Ltp. plantarum* (*L75*)*; Leu. pseudomesenteroides* (*L56*); *Leu. pseudomesenteroides 56 + Ltp. plantarum 75* (*L56 + 75*); LSD—least significant difference; * = *p* ≤ 0.05; ** = *p* ≤ 0.005; *** = *p* ≤ 0.001.

**Table 2:** Changes in colour properties of mango juices as influenced by different lactic acid bacteria.

Mango Cultivar Juice	L*	b*	c*	Δ*ε*
‘Peach’ mango juice				
U 0 h (Raw)	33.62 ± 0.36 ^a^	18.32 ± 0.65 ^a^	18.40 ± 0.65 ^a^	
U 24 h (un-inoculated)	31.38 ± 0.62 ^b^	16.97 ± 0.09 ^b^	17.01 ± 0.11 ^b^	6.09 ± 0.05 ^a^
*L75* 24 h	33.08 ± 0.40 ^a^	18.42 ± 0.89 ^a^	18.63 ± 0.85 ^a^	1.64 ± 0.05 ^k^
*L56* 24 h	33.73 ± 0.34 ^a^	18.46 ± 0.71 ^a^	18.76 ± 0.16 ^a^	1.41 ± 0.05 ^l^
*L56 + 75* 24 h	29.52 ± 0.19 ^b^	15.51 ± 0.45 ^c^	15.63 ± 0.48 ^c^	3.49 ± 0.05 ^f^
‘Sabre’ mango juice				
U 0 h (Raw)	33.04 ± 0.60 ^a^	18.25 ± 1.12 ^a^	18.41 ± 1.14 ^a^	
U 24 h (un-inoculated)	30.46 ± 0.33 ^b^	15.36 ± 0.30 ^d^	15.54 ± 0.31 ^c^	5.64 ± 0.05 ^b^
*L75* 24 h	30.18 ± 0.48 ^b^	14.15 ± 0.44 ^e^	14.32 ± 0.47 ^d^	4.00 ± 0.05 ^d^
*L56* 24 h	30.57 ± 0.19 ^b^	15.98 ± 0.61 ^b,c^	16.08 ± 0.55 ^b,c^	3.41 ± 0.05 ^h^
*L56 + 75* 24 h	30.29 ± 0.27 ^b^	13.52 ± 0.88 ^e^	13.77 ± 0.93 ^d^	5.16 ± 0.05 ^c^
‘Tommy Atkins’ mango juice				
U 0 h (Raw)	27.03 ± 0.66 ^c^	11.12 ± 0.14 ^f^	9.90 ± 0.25 ^e^	
U 24 h (un-inoculated)	25.02 ± 0.13 ^d^	9.16 ± 0.45 ^g^	9.90 ± 0.25 ^e^	3.54 ± 0.05 ^e^
*L75* 24 h	25.00 ± 0.21 ^d^	8.78 ± 0.10 ^g^	9.42 ± 0.12 ^e^	2.65 ± 0.05 ^i^
*L56* 24 h	25.87 ± 0.10 ^d^	9.55 ± 0.10 ^g^	10.44 ± 0.09 ^e^	2.25 ± 0.05 ^j^
*L56 + 75* 24 h	25.82 ± 0.07 ^d^	8.93 ± 0.25 ^g^	9.58 ± 0.27 ^e^	3.43± 0.05 ^g^
LSD	0.19 **	4.65 ***	10.44 ***	0.18 ***

Values with the same alphabetic letter along the column are not significantly different (*p* ≤ 0.05). Keys: U 0 h—raw unfermented mango juice (control); U 24 h—un-inoculated mango juice stored for 24 h; *Ltp. plantarum* (*L75*)*; Leu. pseudomesenteroides* (*L56*); *Leu. pseudomesenteroides 56 + Ltp. plantarum 75* (*L56 + 75*); L*—degree of lightness; b*—degree of blueness; c*—chroma; Δε—colour difference; LSD—least significant difference; ** = *p* ≤ 0.005; *** = *p* ≤ 0.001.

**Table 3:** Changes in antioxidants of mango juices as influenced by different lactic acid bacteria.

Juice of Different Mango Cultivar	Ascorbic Acid (mg/100 g)	TPC (mg/100 mL)	Beta-Carotene (µg/mL)	FRAP (µM TEAC/mL)	DPPH IC50 (mg/mL)	ABTS IC50 (mg/mL)
‘Peach’ mango juice						
U 0 h (control)	10.42 ± 0.06 ^d^	1171.41 ± 0.01 ^f^	3.78 ± 0.22 ^h^	354.19 ± 0.06 ^g^	16.33 ± 0.01 ^n^	10.67 ± 0.11 ^o^
U 24 h (un-inoculated)	5.19 ± 0.06 ^k^	1051.45 ± 0.01 ^k^	10.85 ± 0.83 ^d^	254.94 ± 0.01 ^n^	163.52 ± 0.02 ^a^	28.29 ± 0.06 ^e^
*L75* 24 h	4.49 ± 0.06 ^l^	1393.07 ± 0.01 ^b^	17.02 ± 1.62 ^b^	550.39 ± 0.01 ^b^	95.92 ± 0.01 ^e^	14.51 ± 0.43 ^m^
*L56* 24 h	4.13 ± 0.01 ^m^	1170.07 ± 0.01 ^h^	19.89 ± 1.16 ^a^	330.39 ± 0.01 ^h^	109.51 ± 0.01 ^c^	27.10 ± 1.01 ^f^
*L56 + 75* 24 h	3.87 ± 0.01 ^n^	1364.57 ± 0.01 ^c^	16.33 ± 1.95 ^c^	461.33 ± 0.58 ^d^	104.15 ±0.01 ^d^	22.16 ± 0.01 ^l^
‘Sabre’ mango juice						
U 0 h (control)	15.51 ± 0.01 ^a^	1167.03 ± 0.01 ^i^	0.64 ± 0.52 ^j^	356.91 ± 0.01 ^f^	22.17 ± 0.01 ^m^	24.97 ± 0.01 ^j^
U 24 h (un-inoculated)	10.71 ± 0.01 ^c^	1007.08 ± 0.01 ^m^	4.02 ± 0.19 ^g^	295.06 ± 0.01 ^e^	56.04 ± 0.01 ^i^	43.27 ± 2.17 ^a^
*L75* 24 h	11.23 ± 0.01 ^b^	1415.71 ± 0.01 ^a^	4.32 ± 0.81 ^f^	558.28 ± 0.01 ^a^	44.77 ± 0.01 ^l^	25.27 ± 1.11 ^i^
*L56* 24 h	9.03 ± 0.01 ^g^	1174.64 ± 0.01 ^e^	8.03 ± 1.25 ^e^	390.37 ± 0.06 ^l^	54.96 ± 0.01 ^j^	37.38 ± 6.06 ^d^
*L56 + 75* 24 h	9.29 ± 0.01 ^f^	1211.33 ± 0.01 ^d^	0.97 ± 0.42 ^i^	547.51 ±0.01 ^c^	52.52 ± 0.01 ^k^	27.45 ± 4.33 ^g^
‘Tommy Atkins’ mango juice						
U 0 h (control)	9.68 ± 0.01 ^e^	923.06 ± 0.01 ^n^	0.02 ± 0.00 ^o^	297.74 ± 0.01 ^k^	58.52 ± 0.01 ^h^	13.79 ± 0.91 ^n^
U 24 h (un-inoculated)	8.52 ± 0.01 ^h^	1089.72 ± 0.01 ^j^	0.08 ± 0.01 ^n^	229.74 ± 0.02 ^o^	112.53 ± 0.01 ^b^	40.06 ± 3.51 ^b^
*L75* 24 h	7.87 ± 0.01 ^i^	1170.84 ± 0.01 ^g^	0.12 ±0.00 ^m^	322.07 ± 0.01 ^i^	58.28 ± 0.01 ^h^	23.04 ± 1.24 ^k^
*L56* 24 h	3.61 ± 0.01 ^o^	1046.38 ± 0.01 ^l^	0.15 ± 0.02 ^k^	284.19 ± 0.01 ^m^	62.87 ± 0.01 ^g^	39.86 ± 2.11 ^c^
*L56 + 75* 24 h	7.23 ± 0.01 ^j^	823.31 ± 0.01 ^l^	0.13 ±0.02 ^l^	298.07 ± 0.01 ^j^	63.98 ± 0.01 ^f^	26.86 ± 0.77 ^i^
LSD	3.90 ***	8.019 ***	0.56 ***	1.34 ***	1.27 ***	0.14 ***

Values with the same alphabetic letter along the column are not significantly different (*p* ≤ 0.05). Keys: U 0 h—raw unfermented mango juice (control); U 24 h—un-inoculated mango juice stored for 24 h; *Ltp. plantarum* (*L75*)*; Leu. pseudomesenteroides* (*L56*); *Leu. pseudomesenteroides 56 + Ltp. plantarum 75* (*L56 + 75*); TPC—total phenolic content; FRAP—ferric reducing antioxidant power; DPPH—2,2-diphenyl-1-picrylhydrazyl radial scavenging activity; ABTS—2,2′-azinobis-3-ethylbenzothiazoline-6-sulfonic acid; TEAC—trolox-equivalent antioxidant capacity; LSD—least significant difference; *** = *p* ≤ 0.001.

**Table 4:** Changes in volatile compounds of mango juices as influenced by different lactic acid bacteria.

	‘Peach’					‘Sabre’					‘Tommy Atkins’				
Volatile Compounds	U 0 h	U 24 h	*L75* 24 h	*L56* 24 h	*L56 + 75* 24 h	U 0 h	U 24 h	*L75* 24 h	*L56* 24 h	*L56 + 75* 24 h	U 0 h	U 24 h	*L75* 24 h	*L56* 24 h	*L56 + 75* 24 h
ALKANES															
octane-2-methyl	9.5 ^b^	0.7 ^d^	9.8 ^b^	2.0 ^d^	7.1 ^b,c^	9.5 ^b^	3.7 ^c,d^	18.3 ^a^	7.7 ^b^	1.2 ^d^	ND	0.7 ^d^	9.5 ^b^	ND	ND
decane-3,7-dimethyl	9.1 ^d,e^	7.3 ^d,e,f^	20.3 ^b^	11.4 ^c,d^	8.3 ^d,e^	8.7 ^d,e^	5.9 ^e,f,g^	26.2 ^a^	8.6 ^d,e^	13.9 ^c^	2.7 ^f,g^	1.3 ^g^	15.6 ^c^	6.3 ^e,f^	1.6 ^g^
Nonadecane	2.2 ^e,f,g^	1.2 ^f,g^	17.9 ^a^	12.0 ^b,c^	6.9 ^d,e^	5.6 ^d,e,f^	2.5 ^e,f,g^	18.4 ^a^	14.4 ^a,b^	2.7 ^e,f,g^	2.7 ^e,f,g^	ND	15.7 ^a,b^	ND	7.7 ^c,d^
hexadecane	3.1 ^c^	1.6 ^c^	8.9 ^a,b^	7.7 ^b^	8.2 ^a,b^	3.0 ^c^	3.0 ^c^	10.8 ^a^	2.9 ^c^	ND	1.3 ^c^	1.1 ^c^	1.6 ^c^	1.4 ^c^	2.3 ^c^
pentadecane-8-hexyl	7.8 ^c,d^	0.8 ^d^	22.1 ^b^	8.2 ^c,d^	10.9 ^c^	8.6 ^c,d^	ND	60.9 ^a^	4.3 ^e^	1.0 ^e^	4.5 ^d,e^	ND	8.9 ^c,d^	1.3 ^e^	ND
heptane-2,6-dimethyl (piney *aroma*)	0.3 ^e^	0.7 ^e^	3.4 ^b^	0.9 ^e^	0.7 ^e^	2.3 ^c^	1.5 ^d^	4.4 ^a^	2.6 ^c^	0.55 ^e^	0.9 ^e^	0.5 ^e^	0.4 ^e^	0.5 ^e^	0.3 ^e^
KETONES															
2-pentanone (fruity)	ND	ND	ND	ND	ND	ND	ND	6.3 ^a^	ND	ND	ND	ND	ND	ND	ND
2-heptanone (fruity/floral)	ND	ND	ND	ND	ND	ND	ND	7.6 ^a^	ND	ND	ND	ND	ND	ND	ND
ESTERS															
butyl isobutyrate (fruity type)	ND	ND	10.3 ^b^	ND	ND	1.8 ^d^	ND	15.5 ^a^	ND	0.1 ^e,f^	ND	ND	1.8 ^c,d^	1.6 ^d,e^	ND
butyl acetate (banana or apple)	ND	ND	ND	ND	ND	ND	1.2 ^b^	22.6 ^a^	ND	ND	ND	ND	ND	ND	ND
amyl acetate (fresh-fruity, reminiscent of pear, banana and apple)	ND	ND	ND	ND	ND	ND	ND	0.3 ^a^	ND	ND	ND	ND	ND	ND	ND
ethyl hexanoate (fruity type)	ND	ND	12.4 ^a^	ND	ND	ND	ND	ND	ND	ND	ND	ND	ND	ND	ND
methyl acetate (fruity)	ND	ND	4.7 ^a^	ND	0.6 ^b^	ND	ND	ND	ND	ND	ND	ND	ND	ND	ND
ethyl acetate (fruity)	ND	ND	9.5 ^a^	5.7 ^b^	ND	ND	ND	ND	ND	ND	ND	ND	ND	ND	ND
2,6-di-t-butyl-methylphenol acetate	56.5 ^b^	14.0 ^d^	83.6 ^a^	57.4 ^b^	30.3 ^c,d^	18.6 ^d^	20.1 ^d^	84.9 ^a^	48.1 ^b,c^	22.5 ^d^	19.1 ^d^	18.9 ^d^	57.4 ^b^	34.2 ^c,d^	29.0 ^c,d^
ethyl butyrate (fruity)	24.0 ^c,d^	16.4 ^f^	49.0 ^a^	43.4 ^a^	46.7 ^a^	31.8 ^b^	18.0 ^e,f^	49.0 ^a^	44.7 ^a^	47.7 ^a^	ND	21.7 ^d,e,f^	33.4 ^b^	22.0 ^d,e^	28.7 ^b,c^
ethyl octanoate	3.28 ^h^	2.6 ^h^	21.3 ^d^	13.6 ^f^	13.3 ^f^	12.5 ^f^	5.5 ^g^	30.6 ^a^	26.6 ^b^	24.3 ^c^	2.6 ^h^	ND	17.9 ^e^	2.7 ^h^	13.6 ^f^
isobutyl acetate (waxy type)	5.5 ^d^	ND	4.8 ^d,e^	2.9 ^e^	3.3 ^e^	11.8 ^c^	ND	16.9 ^a^	14.1 ^b^	14.1 ^b^	ND	ND	3.9 ^d,e^	2.9 ^e^	4.0 ^d,e^
ethyl decanoate (waxy type)	2.9 ^c^	ND	5.2 ^b^	ND	ND	ND	ND	8.9 ^a^	5.2 ^b^	8.2 ^a^	ND	ND	4.3 ^b^	ND	ND
methyl benzoate (floral)	ND	ND	ND	ND	ND	ND	ND	3.8 ^a^	ND	ND	ND	ND	ND	ND	ND
geranyl isovalerate (floral type, green)	ND	ND	ND	ND	ND	0.9 ^b^	ND	1.3 ^a^	1.0 ^a,b^	0.9 ^b^	ND	ND	ND	ND	ND
linalyl butyrate (floral type)	ND	ND	1.5 ^c^	ND	0.9 ^d^	ND	ND	2.3 ^a^	ND	1.9 ^b^	ND	ND	0.9 ^d^	ND	0.7 ^d^
ALDEHYDES															
nonanal (rose-orange)	ND	ND	ND	ND	ND	ND	ND	2.67 ^a^	ND	ND	ND	ND	ND	ND	ND
e-2 hexenal (green)	2.9 ^e,f^	0.9 ^f,g^	15.6 ^a,b^	1.2 ^f,g^	5.9 ^c,d^	0.9 ^f,g^	1.1 ^f,g^	17.6 ^a^	1.2 ^f,g^	7.2 ^c^	1.2 ^f,g^	ND	14.2 ^b^	4.6 ^d,e^	3.6 ^e^
pentanal (fruity)	ND	ND	25.9 ^a^	ND	ND	ND	ND	25.9 ^a^	0.04 ^d^	20.7 ^b^	ND	ND	0.04 ^d^	3.9 ^c^	0.03 ^d^
decanal (green)	ND	ND	1.7 ^b^	ND	ND	ND	ND	2.7 ^a^	ND	ND	ND	ND	ND	ND	ND
ALCOHOL															
e-3 hexanol (green)	27.3 ^c,d^	13.9 ^e^	33.9 ^a,b^	27.3 ^c,d^	30.3 ^b,c^	27.3 ^c,d^	14.6 ^e^	37.3 ^a^	29.6 ^b,c^	37.3 ^a^	23.3 ^d^	11.9 ^e^	30.6 ^b,c^	27.9 ^c,d^	27.6 ^c,d^
penten-3-ol (fruity, and green)	13.3 ^c,d^	13.4 ^c,d^	23.3 ^b^	14.1 ^c,d^	18.0 ^b,c^	20.4 ^b^	17.4 ^b,c^	36.7 ^a^	22.7 ^b^	22.7 ^b^	20.5 ^b^	8.7 ^d^	21.4 ^b^	18.7 ^b,c^	20.3 ^b^
1-dodecanol (floral, soapy, waxy)	ND	ND	ND	ND	ND	2.40 ^b^	ND	5.8 ^a^	2.8 ^b^	5.7 ^a^	ND	ND	ND	ND	ND
TERPENE HYDROCARBONS															
β-myrcene (earthy, fruity, and clove-like)	4.1 ^d,e^	2.9 ^e^	9.1 ^c^	5.4 ^d,e^	5.4 ^d,e^	3.4 ^d,e^	1.9 ^e^	25.4 ^a^	5.4 ^d,e^	16.0 ^b^	5.4 ^e^	3.0 ^e^	7.0 ^c,d^	5.4 ^d,e^	5.4 ^d,e^
TOTAL VOLATILE COMPOUNDS	171.7	76.3	388.9	213.1	196.7	169.2	96.3	543.0	541.9	248.6	84.1	67.8	244.4	133.4	144.8

Values with the same alphabetic letter along the column are not significantly different (*p* ≤ 0.05). Keys: U 0 h—raw unfermented mango juice (control); U 24 h—un-inoculated mango juice stored for 24 h; *Ltp. plantarum* (*L75*)*; Leu. pseudomesenteroides* (*L56*); *Leu. pseudomesenteroides 56 + Ltp. plantarum 75* (*L56 + 75*); not detected (ND).

## Data Availability

The datasets generated for this study are available on request to the corresponding author.

## Supplementary Materials

The following supporting information can be downloaded at: https://www.mdpi.com/article/10.3390/foods11050682/s1, Table S1: Definition of attributes and references for the sensory evaluation of mango juice; Table S2A–C: Microbial counts in lactic acid bacteria fermented and unfermented ‘Peach’, ‘Sabre’ and ‘Tommy Atkins’ mango juices respectively.
