# Supplementary material for: Influence of Lactic Acid Bacterium Strains on Changes in Quality, Functional Compounds and Volatile Compounds of Mango Juice from Different Cultivars during Fermentation
Source: Foods. 2022 Feb 25;11(5):682. doi: 10.3390/foods11050682 (PMC8909300; doi:10.3390/foods11050682)
Supplement: Supplementary file 1 [file foods-11-00682-s001.zip › foods-1605126sup.pdf]

Table S1: Definition of attributes and references for the sensory evaluation of mango juice

S/N	Attributes	Description	References	Intensity
1	Colour	Perception of bright yellow of juice (5 to 10)<br>Perception of dark colour of juice (0 to 4)	Bright : whole fresh mango juice<br><br>Dark: Fresh mango juice + 0.6% food grade browning	Absent = 0<br>Dark yellow = 1-4<br>Average = 5-6<br>Bright yellow = 7-10
2	Mango aroma /flavour	Characteristics aroma taste of mango (0 to 10)	Characteristic: Undiluted fresh mango juice<br>Non-characteristics: undiluted mango juice stored at 55 ^{o}C for 24 days.	Absent =0<br>Weak = 1-4<br>Moderate = 5-6<br>Strong = 7-10
3	Consistency	Perception of viscosity product in the mouth (0 to 10)	Weak = 1% diluted mango juice<br>Moderate = 50% diluted mango juice<br>Strong = 100% Fresh mango juice	Absent =0<br>Weak = 1-4<br>Moderate = 5-6<br>Strong = 7-10
4	Sweetness	Perception of sweet taste on the tongue (0 to 10)	Sucrose solution in water.<br>Weak = 2% sucrose solution<br>Moderate = 5% sucrose solution<br>Strong = 15% sucrose solution	Absent = 0<br>Weak = 1-4<br>Moderate = 5-6<br>Strong = 7-10
5	Bitterness	Perception of bitterness associated with caffeine or quinine solutions (0 to 10)	Caffeine acid solution in water.<br>Weak = 0.5% caffeine solution<br>Moderate = 1% caffeine solution<br>Strong = 5% caffeine solution	Absent = 0<br>Weak = 1-4<br>Moderate = 5-6<br>Strong = 7-10
6	Sourness	Perception of pungent, slightly sour characteristics of fermented fruits in the mouth or<br>Perception of acid or fermented taste on the tongue (0 to 10).	Citric acid solution in water.<br>Weak = 0.5% citric acid solution<br>Moderate = 0.1% citric acid solution<br>Strong = 0.5% citric acid solution	Absent = 0<br>Weak = 1-4<br>Moderate = 5-6<br>Strong = 7-10

7	Acceptability	General acceptable attributes for a fermented product.	Fermented mango juice from each cultivar	Absent =0<br>Weak = 1-4<br>Moderate = 5-6<br>Strong = 7-10

Table S2A. Microbial counts in lactic acid bacteria fermented and unfermented 'Peach' mango juices

'Peach' mango juice	*Salmonella* spp.	*Shigella* spp.	*E. coli*	Coliform	Yeast and mold	Total aerobic bacteria
**PU 0 h**	nd	nd	nd	nd	nd	nd
**PU 2 h**	nd	nd	nd	nd	nd	nd
**PU 24 h**	nd	nd	nd	nd	0,2 ± 0,01 h	3,5 ± 0.1 f
**PU 48 h**	nd	nd	nd	nd	0,1 ± 0,01 hi	2,8 ± 0.1 g
**PU72 h**	nd	nd	nd	nd	0,02 ± 0.1 i	1,7 ± 0.1 i
**L75 0 h**	nd	nd	nd	nd	nd	nd
**L75 2 h**	nd	nd	nd	nd	nd	8,26 ± 0,1 b
**L75 24 h**	nd	nd	nd	nd	4,43 ± 0.11 a	9,33 ± 0.1 a
**L75 48 h**	nd	nd	nd	nd	3,33 ± 0.11 c	7,53 ± 0.1 c
**L75 72 h**	nd	nd	nd	nd	2,56 ± 0.05 d	4,56 ± 0.1 e
**L56 0 h**	nd	nd	nd	nd	nd	nd
**L56 2 h**	nd	nd	nd	nd	nd	6,56 ± 0.1 d
**L56 24 h**	nd	nd	nd	nd	4,16 ± 0.05 a	8,33 ± 0,1 b
**L56 48 h**	nd	nd	nd	nd	3,33 ± 0.11 c	4,53 ± 0.1 e
**L56 72 h**	nd	nd	nd	nd	1,6 ± 0.05 e	2,53 ± 0.1 h
**L56+L75 0 h**	nd	nd	nd	nd	nd	nd
**L56+L75 2 h**	nd	nd	nd	nd	nd	4,56 ± 0.1 e
**L56+L75 24 h**	nd	nd	nd	nd	3,3 ± 0.17 c	6,43 ± 0.1 d
**L56+L75 48 h**	nd	nd	nd	nd	1,46 ± 0.05 f	2,43 ± 0.1 h
**L56+L75 72 h**	nd	nd	nd	nd	0,56 ± 0.05 g	1,6 ± 0.1 i
**LSD**					0.16 **	0.01**

Values with the same alphabetic letter along the column are not significantly different ($p \leq 0.05$). Keys : U 0 h : Raw unfermented mango juice (control), U 24 h : Un-inoculated mango juice stored for 24 h ; *Ltp. plantarum* (*L75*); *Leu. pseudomesenteroides* (*L56*); *Leu. pseudomesenteroides 56* + *Ltp. plantarum 75* (*L56+75*); not detected (nd).

Table S2B. Microbial counts in lactic acid bacteria fermented and unfermented 'Sabre' mango juices

'Sabre' mango juice	*Salmonella* spp.	*Shigella* spp.	*E. coli*	*Coliform*	Yeast and mold	Total aerobic bacteria
**SU 0 h**	nd	nd	nd	nd	nd	nd
**SU 2 h**	nd	nd	nd	nd	nd	nd
**SU 24 h**	nd	nd	nd	nd	0,3 ± 0.1 h	4,36 ± 0.23 g
**SU 48 h**	nd	nd	nd	nd	0,23 ± 0.1 h	3,76 ± 0.05 h
**SU72 h**	nd	nd	nd	nd	0,02 ± 01 i	2,73 ± 0.05 j
**L75 0 h**	nd	nd	nd	nd	nd	nd
**L75 2 h**	nd	nd	nd	nd	nd	8,63 ± 0.05 c
**L75 24 h**	nd	nd	nd	nd	5,43 ± 0.11 a	9,83 ± 0.05 a
**L75 48 h**	nd	nd	nd	nd	4,36 ± 0.05 c	8,46 ± 0.05 d
**L75 72 h**	nd	nd	nd	nd	3,56 ± 0.05 d	5,46 ± 0.23 f
**L56 0 h**	nd	nd	nd	nd	nd	nd
**L56 2 h**	nd	nd	nd	nd	nd	7,53 ± 0.1 e
**L56 24 h**	nd	nd	nd	nd	5,16 ± 0.05 b	9,33 ± 0.1 b
**L56 48 h**	nd	nd	nd	nd	4,36 ± 0.05 c	5,53 ± 0.05 d
**L56 72 h**	nd	nd	nd	nd	2,63 ± 0.05 e	3,56 ± 0.05 i
**L56+L75 0 h**	nd	nd	nd	nd	nd	nd
**L56+L75 2 h**	nd	nd	nd	nd	nd	5,53 ± 0.11 f
**L56+L75 24 h**	nd	nd	nd	nd	4,36 ± 0.05 c	7,43 ± 0.05 e
**L56+L75 48 h**	nd	nd	nd	nd	2,46 ± 0.05 f	3,43 ± 0.11 i
**L56+L75 72 h**	nd	nd	nd	nd	1,63 ± 0.05 g	2,56 ± 0,05 k
**LSD**					0.08 **	0.01**

Values with the same alphabetic letter along the column are not significantly different ($p \leq 0.05$). Keys : U 0 h : Raw unfermented mango juice (control), U 24 h : Un-inoculated mango juice stored for 24 h ; *Ltp. plantarum* (*L75*); *Leu. pseudomesenteroides* (*L56*); *Leu. pseudomesenteroides 56* + *Ltp. plantarum 75* (*L56+75*); not detected (nd).

Table S2C. Microbial counts in lactic acid bacteria fermented and unfermented 'Tommy Atkins' mango juices

'Tommy Atkins' mango juice	*Salmonella* spp.	*Shigella* spp.	*E. coli*	Coliform	Yeast and mold	Total aerobic bacteria
**TU 0 h**	nd	nd	nd	nd	nd	nd
**TU 2 h**	nd	nd	nd	nd	nd	nd
**TU 24 h**	nd	nd	nd	nd	0,10 ± 0.05i	2,46 ± 0.05 i
**TU 48 h**	nd	nd	nd	nd	0,08 ± 0.05 i	1,53 ± 0.05 k
**TU72 h**	nd	nd	nd	nd	0,01 ± 0.05 j	0,63 ± 0.01 l
**L75 0 h**	nd	nd	nd	nd	nd	nd
**L75 2 h**	nd	nd	nd	nd	nd	7,56 ± 0.5 b
**L75 24 h**	nd	nd	nd	nd	3,46 ± 0.05 a	8,4 ± 0.1 a
**L75 48 h**	nd	nd	nd	nd	2,36 ± 0.05 d	6,46 ± 0.1 d
**L75 72 h**	nd	nd	nd	nd	1,56 ± 0.05 e	3,56 ± 0.1 g
**L56 0 h**	nd	nd	nd	nd	nd	nd
**L56 2 h**	nd	nd	nd	nd	nd	5,56 ± 0.01 e
**L56 24 h**	nd	nd	nd	nd	3,23 ± 0.05 b	7,33 ± 0.01 c
**L56 48 h**	nd	nd	nd	nd	2,43 ± 0.05 c	3,46 ± 0.01 h
**L56 72 h**	nd	nd	nd	nd	0,63 ± 0.05 f	1,56 ± 0.01 k
**L56+L75 0 h**	nd	nd	nd	nd	nd	nd
**L56+L75 2 h**	nd	nd	nd	nd	nd	3,63 ± 0.05 g
**L56+L75 24 h**	nd	nd	nd	nd	2,43 ± 0.05 c	4,36 ± 0.05 f
**L56+L75 48 h**	nd	nd	nd	nd	0,53 ± 0.05 g	1,76 ± 0.05 j
**L56+L75 72 h**	nd	nd	nd	nd	0,36 ± 0.05 h	0,7 ± 0.05 l
**LSD**					0.06**	0.01**

Values with the same alphabetic letter along the column are not significantly different ($p \leq 0.05$). Keys : U 0 h : Raw unfermented mango juice (control), U 24 h : Un-inoculated mango juice stored for 24 h ; *Ltp. plantarum* (*L75*); *Leu. pseudomesenteroides* (*L56*); *Leu. pseudomesenteroides 56* + *Ltp. plantarum 75* (*L56+75*); not detected (nd).
